# The value of hip circumference/height^x^ ratio for identifying childhood hypertension

**DOI:** 10.1038/s41598-018-21676-4

**Published:** 2018-02-19

**Authors:** Na Lu, Rui Wang, Meijing Ji, Xiaoli Liu, Lu Qiang, Chunming Ma, Fuzai Yin

**Affiliations:** Department of Endocrinology, The First Hospital of Qinhuangdao, Qinhuangdao, 066000 Hebei Province China

## Abstract

To investigate the value of hip circumference related indexes for identifying childhood hypertension. In 2011, 1,352 Han children aged 7–12 years were recruited in our study. Hypertension was defined as systolic blood pressure or diastolic blood pressure ≥95th percentile for all three screenings. We set the power value of the hip circumference/height^x^ ratio (x = 0, 0.8, 1 and 1.5) and studied the association with blood pressure. Hip circumference, hip circumference/height^0.8^, hip circumference/height and hip circumference/height^1.5^ all showed a positive correlation with systolic blood pressure and diastolic blood pressure(*P* < 0.05). Area under the curve (AUC) was used to evaluate the abilities of hip circumference related indexes. Hip circumference/height^0.8^, hip circumference/height and hip circumference/height1.5 were not superior to hip circumference. The present study demonstrates that hip circumference measurement is a helpful tool to detect the presence of hypertension in Han children 7–12 years old.

## Introduction

In recent decades, childhood hypertension has become an important health issue due to its rising prevalence and its risk for end-organ damage^1,2^. Obesity is a growing problem throughout the world, recently it also tends to be younger. Clinical data showed obesity is closely correlated with hypertension in children. Obesity is associated with salt sensitivity, dyslipidemia and insulin resistance. These factors increase the risk of hypertension^3^.

In previous studies, body mass index(BMI) and waist circumference(WC) were often used to evaluate obese status. In 2011, a hip circumference related indicator, body adiposity index (BAI), was proposed as a good marker for adiposity in adults^4^. BAI is calculated using the following equation: BAI = hip circumference(cm)/height(m)^1.5^– 18. In 2013, BAI for pediatrics (BAIp): hip circumference(cm)/height(m)^0.8^– 38 was also proposed to predict body fatness in 5–12 years old children^5^. The purposes of this study were to determine the value of hip circumference(cm)/height(m)^x^ ratio for identifying childhood hypertension.

## Methods

### Subjects and Anthropometric Measurements

After obtaining informed consent from all children and their legal guardians, the study was conducted. A total of 1,352 Han children (679 boys and 673 girls) were recruited in the study population. Height, weight, WC, hip circumference and blood pressure were measured. The details of study population and anthropometric measurements were described in our previously published paper^6^. The experiment was approved by the ethics committee of the First Hospital of Qinhuangdao. All methods were performed in accordance with the relevant guidelines and regulations by including a statement in the methods section.

Hip circumference was measured at the level of maximum extension of the hip. Three hip circumference related indexes were calculated by height and hip circumference: hip circumference(cm)/height(m)^0.8^(BAIp), hip circumference(cm)/height(m) and hip circumference(cm)/height(m)^1.5^(BAI).

### Definition of hypertension

All subjects with SBP/DBP ≥95th percentile had repeated measurements on two subsequent occasions. Hypertension was determined as SBP or DBP ≥95th percentile for all three screenings^7^.

### Statistical Analyses

Quantitative data were showed as mean ± standard deviation. Student *t* test and Pearson correlation were used. The area under the curve (AUC) for the hip circumference(cm)/height(m)^x^ ratio was drawn by receiver operating characteristic (ROC) analysis. AUC was used to evaluate the abilities of hip circumference(cm)/height(m)^x^ ratio for identifying hypertension in children. The comparisons of AUC were performed with MedCalc 11.4.2.0 software (Ostend, Belgium). Multiple linear regression analyses were performed to examine the relationships between blood pressure and other variables(sex, age, height, WC and hip circumference). Six models were used, Model 1: sex and age; Model 2: sex and height; Model 3: sex, age and height; Model 4: sex, age, height and WC; Model 5: sex, age, height and hip circumference; Model 6: sex, age, height, WC and hip circumference. Multiple linear regression analyses were also performed to examine the relationships between hip circumference and other variables(sex, age, height and WC). Four models were used, Model 1: sex and age; Model 2: sex and height; Model 3: sex, age, and height; Model 4: sex, age, height and WC. SPSS 11.5 statistical software (SPSS 11.5 for Windows; SPSS, Inc., Chicago, IL) was used for data analysis. *P* < 0.05 was considered statistically significant.

## Results

36 children(16 boys and 20 girls) were diagnosed as hypertension. Anthropometric parameters were shows in Table 1. Compared with girls, the levels of hip circumference, hip circumference/height^0.8^, hip circumference/height and hip circumference/height^1.5^ were higher in boys (*P* < 0.001). As shown in Table 2, age, height, BMI, waist circumference, hip circumference, hip circumference/height^0.8^, hip circumference/height and hip circumference/height^1.5^ positively correlated with systolic blood pressure(SBP) and diastolic blood pressure(DBP), boys and girls both(*P* < 0.05).

**Table 1:** Clinical characteristics of the study subjects.

Variable	Boys (n = 679)	Girls (n = 673)	*t*	*P*
Age(year)	9.4 ± 1.7	9.5 ± 1.6	0.664	0.507
Height(cm)	141.5 ± 11.9	140.7 ± 11.8	1.315	0.189
BMI(kg/m^2^)	19.9 ± 4.6	18.4 ± 3.8	6.559	0.000
WC (cm)	67.8 ± 12.2	63.3 ± 9.7	7.407	0.000
Hip (cm)	79.0 ± 10.9	77.5 ± 10.2	2.534	0.011
Hip/height^0.8^	59.7 ± 5.9	58.9 ± 5.0	2.800	0.005
Hip/height	55.7 ± 5.3	55.0 ± 4.4	2.734	0.006
Hip/height^1.5^	46.9 ± 4.5	46.4 ± 3.7	2.127	0.034
SBP(mmHg)	107.1 ± 11.8	106.9 ± 12.2	0.393	0.694
DBP(mmHg)	66.5 ± 10.1	67.7 ± 10.1	2.267	0.024

BMI: body mass index; WC: waist circumference; SBP: systolic blood pressure; DBP: diastolic blood pressure. Values are expressed as mean ± standard deviation. Comparisons were done between the two groups using the Student *t* test.

**Table 2:** Relationship between obese indexes and blood pressure by gender.

Variable	Total(n = 1352)				Boys(n = 679)				Girls(n = 673)			
	SBP		DBP		SBP		DBP		SBP		DBP	
	*r*	*P*	*r*	*P*	*r*	*P*	*r*	*P*	*r*	*P*	*r*	*P*
Age(year)	0.259	<0.001	0.188	<0.001	0.205	<0.001	0.146	<0.001	0.313	<0.001	0.231	<0.001
Height(cm)	0.352	<0.001	0.219	<0.001	0.340	<0.001	0.185	<0.001	0.364	<0.001	0.260	<0.001
BMI(kg/m^2^)	0.459	<0.001	0245	<0.001	0.489	<0.001	0.230	<0.001	0.441	<0.001	0.299	<0.001
WC (cm)	0.444	<0.001	0.238	<0.001	0.499	<0.001	0.241	<0.001	0.400	<0.001	0.277	<0.001
Hip (cm)	0.486	<0.001	0.284	<0.001	0.496	<0.001	0.244	<0.001	0.477	<0.001	0.338	<0.001
Hip/height^0.8^	0.442	<0.001	0.251	<0.001	0.453	<0.001	0.213	<0.001	0.436	<0.001	0.310	<0.001
Hip/height	0.400	<0.001	0.224	<0.001	0.413	<0.001	0.191	<0.001	0.391	<0.001	0.280	<0.001
Hip/height^1.5^	0.229	<0.001	0.120	<0.001	0.259	<0.001	0.111	0.004	0.196	<0.001	0.143	<0.001

BMI: body mass index; WC: waist circumference; SBP: systolic blood pressure; DBP: diastolic blood pressure.

AUCs were used to evaluate the abilities of hip circumference related indexes for identifying hypertension (Table 3). The AUCs of the hip circumference were higher than the AUCs of the age (boys: z = 2.614, *P* = 0.008; girls: z = 2.485, *P* = 0.013). The AUCs of the hip circumference were slightly higher than the AUCs of the height, but the difference was not significant (boys: z = 1.796, *P* = 0.072; girls: z = 1.841, *P* = 0.065). The AUCs of hip circumference were similar between the AUCs of BMI (boys: z = 0.293, *P* = 0.769; girls: z = 0.044, *P* = 0.964), WC (boys: z = 0.403, *P* = 0.686; girls: z = 0.401, *P* = 0.688), Hip circumference/height^0.8^ (boys: z = 0.752, *P* = 0.452; girls: z = 0.064, *P* = 0.949) and hip circumference/height (boys: z = 1.194, *P* = 0.232; girls: z = 0.286, *P* = 0.774). The AUC of the hip circumference was higher than the AUC of the hip circumference/height^1.5^ in boys, but not in girls (boys: z = 2.453, *P* = 0.014; girls: z = 1.248, *P* = 0.212). The AUCs of hip circumference/height^1.5^ were similar between the AUCs of Hip circumference/height^0.8^ (boys: z = 1.850, *P* = 0.064; girls: z = 1.196, *P* = 0.263) and hip circumference/height (boys: z = 1.425, *P* = 0.154; girls: z = 0.887, *P* = 0.375). The AUC of the hip circumference/height^1.5^ was lower than the AUC of the BMI (boys: z = 2.142, *P* = 0.032; girls: z = 1.152, *P* = 0.249) and WC (boys: z = 2.690, *P* = 0.007; girls: z = 0.813, *P* = 0.416) in boys, but not in girls. The AUCs of BMI, WC, hip circumference/height ratio and hip circumference/height^0.8^ were similar in both genders(*P* > 0.05). In boys, hip circumference cutoff point was 90.4 cm(sensitivity 87.5% and specificity 85.8%). In girls, hip circumference cutoff point was 82.2 cm(sensitivity 85.0% and specificity 71.6%).

**Table 3:** Area under the curves for obese indexes associated with hypertension in boys and girls.

Variable	Boys(n = 679)			Girls(n = 673)		
	AUC (95% CI)	Std.Error	*P*	AUC (95% CI)	Std.Error	*P*
Age(year)	0.728(0.600~0.856)	0.065	0.002	0.668(0.561~0.775)	0.054	0.010
Height(cm)	0.805(0.701~0.909)	0.053	<0.001	0.720(0.625~0.814)	0.048	0.001
BMI	0.898(0.827~0.968)	0.036	<0.001	0.838(0.732~0.943)	0.054	<0.001
WC	0.925(0.880~0.969)	0.023	<0.001	0.809(0.709~0.908)	0.051	<0.001
Hip	0.911(0.859~0.962)	0.026	<0.001	0.835(0.756~0.913)	0.040	<0.001
Hip/height^0.8^	0.877(0.804~0.951)	0.037	<0.001	0.831(0.736~0.925)	0.048	<0.001
Hip/height	0.850(0.763~0.937)	0.044	<0.001	0.816(0.713~0.919)	0.053	<0.001
Hip/height^1.5^	0.737(0.608~0.866)	0.066	0.001	0.745(0.628~0.863)	0.060	<0.001

AUC: area under the curve; BMI: body mass index; WC: waist circumference.

When SBP and DBP were considered as the dependent variables in a multiple regression analysis with sex, age, height, WC and hip circumference as independent variables, the hip circumference maintained an independent association with SBP (*β* = 0.546, 95%CI: 0.376~0.716, *P* < 0.001) and DBP (*β* = 0.307, 95%CI: 0.150~0.464, *P* < 0.001) (Table 4). When hip circumference was considered as the dependent variables in a multiple regression analysis with sex, age, height and WC as independent variables, the age (*β* = 0.274, 95%CI: 0.095~0.454, *P* = 0.003), height (*β* = 0.274, 95%CI: 0.245~0.303, *P* < 0.001) and WC (*β* = 0.679, 95%CI: 0.659~0.699, *P* < 0.001) maintained an independent association with hip circumference (Table 5).

**Table 4:** Multiple linear regression analyses for blood pressure (Enter Method).

Model	SBP			DBP		
	Unstandardized Coefficients B (95% CI)	Standardized Coefficients B	*P*	Unstandardized Coefficients B (95% CI)	Standardized Coefficients B	*P*
Model 1	R^2^ = 0.067			R^2^ = 0.039		
(Constant)	89.634(86.033~93.234)		<0.001	55.821(52.742~58.899)		<0.001
Sex(boys = 0, girls = 1)	−0.369(−1.609~0.870)	−0.015	0.559	1.178(0.118~2.238)	0.058	0.029
Age(year)	1.846(1.478~2.214)	0.259	<0.001	1.126(0.811~1.441)	0.187	<0.001
Model 2	R^2^ = 0.124			R^2^ = 0.053		
(Constant)	56.813(49.608~64.017)		<0.001	39.800(33.491~46.110)		<0.001
Sex(boys = 0, girls = 1)	0.045(−1.157~1.247)	0.002	0.941	1.407(0.354~2.460)	0.070	0.009
Height(cm)	0.356(0.305~0.406)	0.352	<0.001	0.189(0.144~0.233)	0.222	<0.001
Model 3	R^2^ = 0.126			R^2^ = 0.053		
(Constant)	53.490(45.250~61.730)		<0.001	40.819(33.596~48.042)		<0.001
Sex(boys = 0, girls = 1)	0.124(−1.081~1.329)	0.005	0.840	1.383(0.326~2.439)	0.068	0.010
Age(year)	−0.499(−1.100~0.103)	−0.070	0.104	0.153(−0.374~0.680)	0.025	0. 570
Height(cm)	0.413(0.327~0.498)	0.408	<0.001	0.171(0.097~0.246)	0.201	<0.001
Model 4	R^2^ = 0.215			R^2^ = 0.078		
(Constant)	61.487(53.574~69.399)		<0.001	44.398(37.177~51.620)		<0.001
Sex(boys = 0, girls = 1)	1.651(0.483~2.819)	0.069	0.006	2.066(1.001~3.132)	0.102	<0.001
Age(year)	0.112(−0.466~0.690)	0.016	0.704	0.426(−0.101~0.954)	0.071	0.113
Height(cm)	0.121(0.028~0.214)	0.119	0.011	0.041(−0.044~0.126)	0.048	0.349
WC(cm)	0.406(0.341~0.470)	0.382	<0.001	0.182(0.123~0.240)	0.203	<0.001
Model 5	R^2^ = 0.237			R^2^ = 0.088		
(Constant)	66.231(58.330~74.133)		<0.001	46.838(39.561~54.115)		<0.001
Sex(boys = 0, girls = 1)	0.574(−0.554~1.701)	0.024	0.318	1.595(0.557~2.634)	0.079	0.003
Age(year)	−0.061(−0.626~0.504)	−0.009	0.832	0.360(−0.161~0.880)	0.060	0.176
Height(cm)	−0.034(−0.135~0.067)	−0.034	0.511	−0.040(−0.133~0.053)	−0.047	0.404
Hip(cm)	0.586(0.504~0.668)	0.518	<0.001	0.277(0.201~0.352)	0.290	<0.001
Model 6	R^2^ = 0.237			R^2^ = 0.088		
(Constant)	66.058(58.128~73.988)		<0.001	46.970(39.666~54.273)		<0.001
Sex(boys = 0, girls = 1)	0.675(−0.515~1.866)	0.028	0.266	1.518(0.421~2.614)	0.075	0.007
Age(year)	−0.038(−0.610~0.534)	−0.005	0.897	0.342(−0.185~0.869)	0.057	0.203
Height(cm)	−0.029(−0.132~0.074)	−0.029	0.583	−0.044(−0.138~0.051)	−0.051	0.368
WC	0.035(−0.096~0.167)	0.033	0.600	−0.027(−0.148~0.094)	−0.030	0.664
Hip	0.546(0.376~0.716)	0.483	<0.001	0.307(0.150~0.464)	0.322	<0.001

Dependent Variable: SBP or DBP. SBP: systolic blood pressure; DBP: diastolic blood pressure; WC: waist circumference.

**Table 5:** Multiple linear regression analyses for hip circumference (Enter Method).

Model	Unstandardized Coefficients B (95% CI)	Standardized Coefficients B	*P*	R^2^
Model 1				0.328
(Constant)	45.014(42.312~47.715)		<0.001	
Sex(boys = 0, girls = 1)	−1.679(−2.609~−0.749)	−0.079	<0.001	
Age(year)	3.584(3.308~3.860)	0.568	<0.001	
Model 2				0.578
(Constant)	−16.772(−21.190~−12.354)		<0.001	
Sex(boys = 0, girls = 1)	−0.885(−1.623~−0.148)	−0.042	0.019	
Height(cm)	0.677(0.646~0.708)	0.758	<0.001	
Model 3				0.583
(Constant)	−21.750(−26.779~−16.722)		<0.001	
Sex(boys = 0, girls = 1)	−0.768(−1.503~−0.032)	−2.048	0.041	
Age(year)	−0.747(−1.114~−0.380)	−0.118	<0.001	
Height(cm)	0.762(0.710~0.814)	0.853	<0.001	
Model 4				0.904
(Constant)	−8.373(−10.825~−5.921)		<0.001	
Sex(boys = 0, girls = 1)	1.787(1.425~2.149)	0.084	<0.001	
Age(year)	0.274(0.095~0.454)	0.044	0.003	
Height(cm)	0.274(0.245~0.303)	0.307	<0.001	
WC(cm)	0.679(0.659~0.699)	0.723	<0.001	

Dependent Variable: hip circumference. WC: waist circumference.

## Discussion

This study discusses the use of hip circumference related indexes in the evaluation of children hypertension in Han race aged 7–12 years. Hip circumference related indexes were closely related with blood pressure for both boys and girls. Thus, hip circumference related indexes can accurately identify hypertension in Han children.

Increasing evidence has demonstrated that BAI positively correlated with blood pressures in adults, but not superior to BMI^8–11^. BAI and BMI were significant predictors of risk of hypertension and changes in blood pressure after an 8-years follow-up^12^. Dong *et al*. found that BMI could be a better predictor of elevated BP than BAI in Chinese children and adolescents^13^. Comparing with the traditional obese indexes, BMI and WC, BAI was not superior in identifying hypertension, especially in boys.

The result is likely related to the following mechanism. Obesity is key feature in the pathophysiology of hypertension in children. BMI and WC are two common indexes of obesity. BAI is another index for evaluating obesity calculated from measurements of the hip and height. Two studies from china showed that BMI and WC are better tools than BAI for estimating whole body fat and central body fat in Chinese adults and children^14,15^.

BAI was proposed in adults and over-estimated the percentage of body fat in children^5^. El Aarbaoui T *et al*. proposed BAIp. BAIp appears as a new index for children’s body fatness, with acceptable accuracy. Hip circumference/height ratio and BAIp are better markers for overweight (adiposity) in obese children than BAI^16^. However BAIp do not provide better estimate of obese children’s adiposity and metabolic complications than the widely used BMI and WC^16,17^. In our study, the AUCs of hip circumference/height ratio and BAIp were improved than the AUCs of BAI while there is not statistical difference. But the AUCs of BMI, WC, hip circumference/height ratio and BAIp were similar in boys and girls. The result means that the abilities of these indexes were alike for identifying children hypertension. Moreover, all hip circumference related indexes were not superior to hip circumference. In other words, hip circumference related indexes were not improved after adjustment for height and complicated calculation. Hip circumference itself is simple and accuracy.

The determinants of childhood blood pressure include height and adiposity^18,19^. The role of height is a normal growing process. So the diagnostic criteria of the children hypertension is a set of age-, sex- and height-specific criteria of blood pressure. The role of adiposity is a pathological process. Consistent with previous studies^18,19^, the correlation between blood pressure and height was significant after age and sex adjustment and weakened after further adjusting for WC. The increase of blood pressure was due to the childhood normal body growth and adiposity.

Interesting, the correlation between height, WC and blood pressure was not significant after adjusting for hip circumference. In our study, multiple linear regression shows that hip circumference was independent related with age, height and waist circumference. At a young age, children not only grow up, but also expand hip circumference. It is a normal growing process. At the same time, many studies found that hip circumference associated with visceral fat area in children^20,21^. It means that hip circumference can reflects childhood normal body growth and adiposity.

The children of the study were Han race. The range of age was 7–12 years old. The ability of hip circumference related indexes for identifying childhood hypertension should be further confirmed in different races and age groups.

In conclusion, hip circumference measurement is a helpful tool to detect the children with hypertension in Han race 7–12 years old. Compared with BAI and BAIp, hip circumference is more simple.
